# Viral suppression of multiple escape mutants by de novo CD8^+ ^T cell responses in a human immunodeficiency virus-1 Infected elite suppressor

**DOI:** 10.1186/1742-4690-8-63

**Published:** 2011-08-03

**Authors:** Karen A O'Connell, Robert W Hegarty, Robert F Siliciano, Joel N Blankson

**Affiliations:** 1 Department of Medicine, Johns Hopkins University School of Medicine, 733 N. Broadway, Baltimore MD 21205, USA; 2 Howard Hughes Medical Institute, Johns Hopkins University School of Medicine, 733 N. Broadway, Baltimore MD 21205, USA

## Abstract

Elite suppressors or controllers (ES) are HIV-1 infected patients who maintain undetectable viral loads without treatment. While HLA-B*57-positive ES are usually infected with virus that is unmutated at CTL epitopes, a single, dominant variant containing CTL escape mutations is typically seen in plasma during chronic infection. We describe an ES who developed seven distinct and rare escape variants at an HLA-B*57-restricted Gag epitope over a five year period. Interestingly, he developed proliferative, *de novo *CTL responses that suppressed replication of each of these variants. These responses, in combination with low viral fitness of each variant, may contribute to sustained elite control in this ES.

## Findings

Elite suppressors (ES) are HIV-1 infected individuals who control viremia naturally, maintaining clinically undetectable levels of virus in the plasma (< 50 copies of HIV-1 RNA/ml) without antiretroviral therapy[1-3]. We have previously shown that HIV-1 sequences amplified from resting CD4^+^T cells of these patients are distinct from those amplified from free virus in the plasma, with CTL escape mutations apparent in the plasma virus but largely absent in proviral DNA [4-6]. We have also shown that the escape mutations present in plasma virus generally do not vary over time. This suggests that virus in ES achieves a balance between fitness and CTL escape which eliminates the development of further nonsynonymous mutations during chronic infection, despite ongoing viral replication [7-9].

The HLA-B*57 and B*5801 alleles are overrepresented in ES and are thought to play a causative role in control of viremia in these patients [1-3]. The TW10 epitope in Gag (amino acids 240-249) is a well-characterized, immunodominant epitope restricted by these alleles. Escape via a T242N mutation frequently occurs early in the majority of HLA-B*57 chronic progressors (CP) [10] and ES [4,11-14]. Compensatory mutations upstream of TW10 in the CypA binding site partially or completely compensate for the negative fitness impact of the T242N mutation [15]. Interestingly, some ES develop rare, alternative mutations in TW10 [4,13], some of which also have dramatic fitness defects [13].

Here we describe an HLA-B*57-positive ES who displayed unprecedented sequence variation at the TW10 epitope while never acquiring the T242N mutation. Seven different TW10 variants were observed in the plasma sequences from this patient over five years, all of which otherwise shared the same *gag *backbone. Only two of the seven variants have been previously documented in the LANL database, and together these two variants were only present in 5 of the 1510 Clade B HIV-1 sequences in the database http://www.hiv.lanl.gov/content/sequence/HIV/COMPENDIUM/2010compendium.html. Using IFN-γ ELISPOT analysis, we previously showed that this patient, ES8, was able to mount a CTL response against each of the variants [6]. To understand the presence of this atypical ongoing evolution, we also determined the impact of these mutations on viral replication capacity and multiple aspects of the CTL response.

### Autologous plasma variants from ES8 contain rare mutations in TW10 and are significantly less fit than proviral variants or laboratory isolates

**Figure 1:**
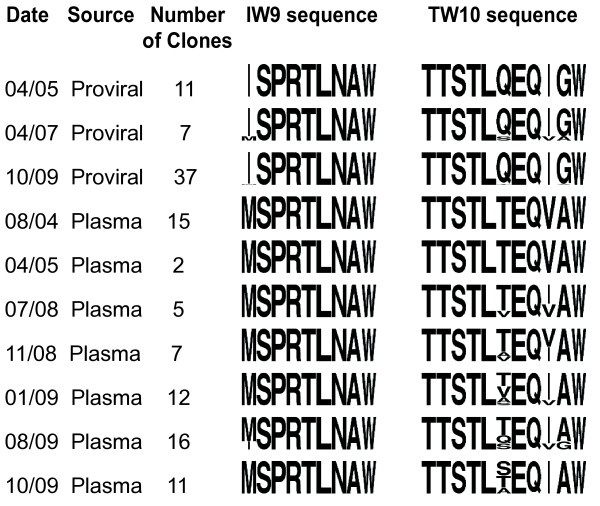
**Proviral and plasma virus variants from ES8 spanning five years**. Clones were amplified using nested, limiting dilution PCR and RT-PCR. Sequence logos were created utilizing the WebLogo program from University of California at Berkeley http://weblogo.berkeley.edu/. For clones amplified at each time point, Logos show the proportion of an amino acid residue and substitutions at a given site. The date the sequence was obtained, the source of the sequence, and the number of clones for each time point are shown, as well as the sequence of the HLA-B*57-restricted IW9 and TW10 epitopes.

Multiple HIV-1 clones were amplified from the plasma of ES8 over the course of five years. To prevent resampling, *gag *genes were amplified from plasma-derived RNA by limiting dilution "digital" nested PCR as previously described [4,11]. Four clones obtained from one time point were identical to the dominant proviral Gag and contained no mutations in HLA-B*57 targeted epitopes. The other 64 Gag clones, however, shared mutations distinguishing them from the dominant proviral variants. These plasma Gag clones were identical to each other with the exception of variation at the TW10 epitope. In total, seven different variations in the TW10 epitope were observed in the plasma Gag sequences (Figure 1). These variants were quite rare in the LANL database, and none of the variants possessed the typical T242N mutation.

**Figure 2:**
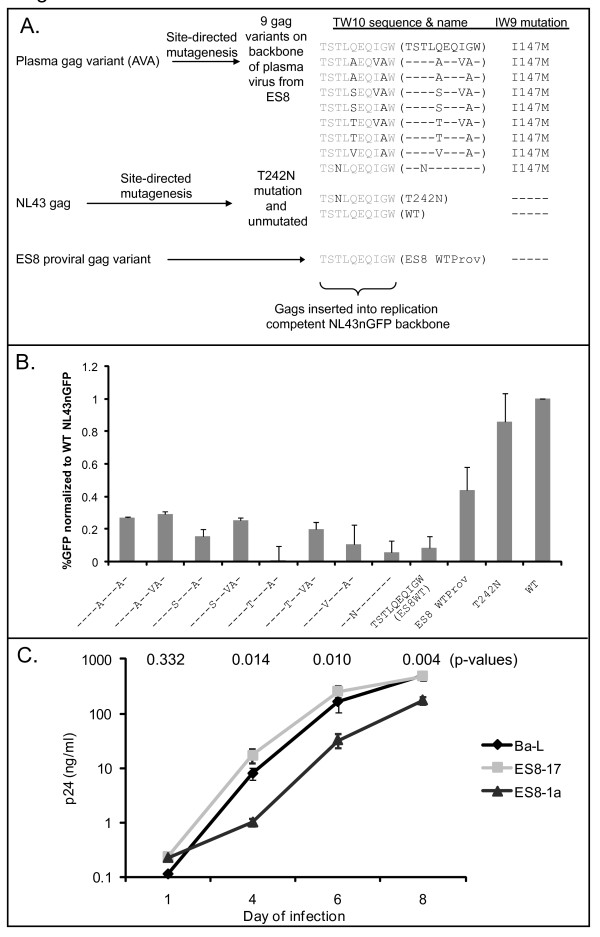
**Mutations present in the TW10 epitope of plasma viral variants dramatically impact viral fitness**. (A) Schematic for the development of NL43nGFP based viruses to test the impact of plasma TW10 variants on viral fitness. *Gag *from ES8-1a virus which contains the TSTLAEQVAW TW10 mutant was mutated using site-directed mutagenesis to produce *gag *with the six other variants present in the plasma, as well as wild type TW10 and T242N on this ES8-1a backbone. Site-directed mutagenesis was also utilized to insert T242N into the NL43 *gag*, and *gag *from ES8-17 was amplified. Each of these *gag *mutants were then inserted into NL43eGFP backbone. All viruses derived from ES8-1a, the viral variant representing plasma viral variants, also had the I147M mutation, as indicated. (B) Fitness of viral variants was measured on day 6 post infection. Data shown are infection of two uninfected donors infected with two separate virus preparations in duplicate. Percent infection was normalized to transfection efficiency, and to the percent infection of WT NL43 which was on average 42%. (C) Replication of viral variants ES8-17 and ES8-1a. ES8-17 is wild type at the TW10 epitope and was cultured from resting CD4^+ ^T cells from ES8. ES8-1a has mutation at TW10 and was cultured from activated CD4^+ ^T cells. Results are the average of triplicate samples of p24 supernatant of activated CD4^+ ^T cells infected with each virus. P-values shown above each time point compare the p24 of ES8-17 and ES8-1a using a one-tailed Student's T-test.

To determine the impact of the mutations on viral replicative capacity (referred to as "fitness" herein), we utilized site-directed mutagenesis to introduce the seven observed plasma *gag *variants into the backbone of one ES8 plasma *gag *clone. As all plasma clones were identical outside of the TW10 epitope, this backbone was representative of all plasma clones. This backbone contained the I147M substitution in the HLA-B*57 restricted IW9 epitope [4], but no escape mutations in other HLA-B*57 restricted Gag epitopes. In addition to mutating TW10 to express each of the seven plasma variants, we mutated the epitope to express the T242N mutation and to express wild type TW10 (TSTLQEQIGW). We then inserted these 9 *gag *variants into an NL43 provial clone in which *nef *has been replaced by GFP (NL43nGFP) to make infection detectable by flow cytometric analysis. We compared the fitness of these nine variants to three control variants: wild type NL43, wild type NL43 with the T242N mutation, and wild type NL43 with *gag *replaced by *gag *of the dominant proviral variant of the patient (Figure 2a). The dominant proviral variant of ES8 had no mutation in any HLA-B*57 restricted epitope. Input virus was standardized by normalizing to transfection efficiency, and fitness was assessed as previously described [16].

As shown previously, the T242N mutation slightly reduced fitness of otherwise wild type NL43nGFP [15,17]. Viruses expressing the I147M mutation in the IW9 epitope and any of the ES8 plasma mutations in TW10, however, showed considerably less infection of donor cells than the control viruses. Virus containing the T242N mutation in the patient's plasma *gag *backbone also showed little replication. Interestingly, reversion of the TW10 mutations seen in the plasma variants to a wild type TW10 motif (ES8WT) did not rescue viral fitness and, if anything, decreased viral fitness further. This may suggest that compensatory mutations exist in the plasma variants which partially rescue fitness defects caused by the TW10 mutations, but that these compensatory mutations reduce the fitness of a virus with wild type TW10. Virus with Gag derived from provirus of ES8 (ES8WTProv) had an intermediate level of fitness (Figure 2b). The proviral-derived Gag had no mutations in either the IW9 or TW10 epitopes, suggesting that mutations in the HLA-B*57 restricted epitopes are not the only factors impacting the fitness of virus from ES8.

To elucidate the relevance of the TW10 mutations in virus obtained directly from the patient, we compared the fitness of two viral variants which we cultured from ES8. ES8-1A and ES8-17 are variants cultured from activated CD4^+^T cells and resting CD4^+^T cells from the patient, respectively [18]. The ES8-1A variant expresses mutant IW9 and TW10 epitopes, specifically the TSTL**A**EQ**VA**W variant, and is thus identical to some plasma viral clones. The ES8-17 virus is unmutated at the TW10 and IW9 epitopes. We infected activated CD4^+^T cells from uninfected donors with equal amounts of each virus via spinoculation [19] in triplicate, as well as the laboratory strain Ba-L (virus quantified using p24 assay), and found that the ES8-17 variant showed similar replication kinetics to the laboratory strain, whereas ES8-1A had reduced replication kinetics (Figure 2c). Together, these studies examining the fitness of viral variants from this patient show that plasma variants were less fit than the ancestral virus which was retained in the proviral compartment and less fit than wild type NL43. The low fitness of the plasma variants relative to proviral suggests that viral fitness is a consequence rather than a cause of elite control in this patient.

### The CD8^+^T cell response to autologous plasma virus is proliferative and de novo

**Figure 3:**
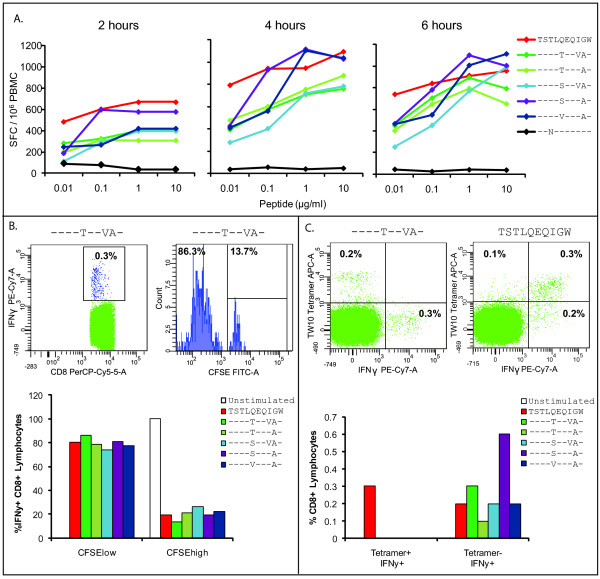
**CD8+ T cells from ES8 mount a proliferative response to autologous TW10 variants**. (A) CD8^+ ^T cells from ES8 mount a strong response to wild type TW10 and autologous variants, but not to TW10 with the T242N mutation. IFN-γ ELISPOT measuring the response of CD8+ T cells from ES8 to wild type TW10, autologous variants, and the T242N mutant. Data is from 2 hours (left), 4 hours (center), and 6 hours (right) of stimulation with the peptides. (B) IFN-γ ^+ ^CD8^+ ^lymphocytes that have been stimulated with wild type TW10 and autologous variants are undergoing proliferation. Representative dot plot and histogram shown for stimulation with one of the autologous mutants, TSTLTEQVAW (top). Gating is on the population of CD8^+ ^T cells that are IFN-γ ^+ ^(bottom). (C) HLA-B*57 tetramer^+ ^cells are producing IFN-γ only in response to wild type TW10 peptide, but not to autologous variants. Representative dot plot and histogram shown for stimulation with one of the autologous mutants, TSTLTEQVAW (top left) and wild type TW10 (top right). Gating is on CD8^+ ^T cells (bottom). Total of 500,000 events were collected for panels B and C, and the peptide with which cells were stimulated indicated in the legend.

To examine the CTL response to the patient's autologous variants, we selected the five TW10 variants which were most highly represented in the plasma and evaluated the ability of CD8^+^T cells from ES8 to respond to these peptides in comparison to WT TW10, or TW10 containing the T242N mutation. Using IFN-γ ELISPOT, we had previously shown that CTL from ES8 exhibited a strong, high-avidity IFN-γ response to all five variants while failing to respond to TW10 with the T242N mutation [6]. We considered the possibility that there would be differences in the kinetics of the IFN-γ response to the autologous epitopes, however, and therefore measured the response to autologous viral variants over a shorter time period, from 2 hours, 4 hours, and 6 hours after exposure to peptide (Figure 3a). We again found comparable IFN-γ responses to the wild type TW10 and the autologous variants and no response to the T242N mutant.

While IFN-γ provides a measure of whether an antigen is recognized by CTL, IFN-γ is not a good measure of an effective CTL response and does not correlate with control of viral replication [20-22]. The capacity of CD8^+^T cells to proliferate in response to HIV-1 antigen, however, is associated with control of HIV-1 viremia [23,24]. CFSE analysis was therefore used to determine whether CTL proliferated in response to the escape mutants. In addition, we looked at CFSE in conjunction with IFN-γ and HLA-B*57 TW10 tetramer staining to elucidate the population of CD8^+^T cells responding to peptide. We found that the vast majority of IFN-γ producing cells were also CFSElow, suggesting that cells producing cytokines in response to peptides were also proliferating (Figure 3b). Interestingly, we found that cells responding to the patient's autologous peptides did not react to the tetramer, and only wild type TW10 elicited a response from tetramer staining cells (Figure 3c). This implies that the response to the patient's autologous peptides was the result of an entirely de novo response rather than cross-reactivity by T cells specific for wild type TW10 peptides.

### CTL from ES8 suppress viral replication

**Figure 4:**
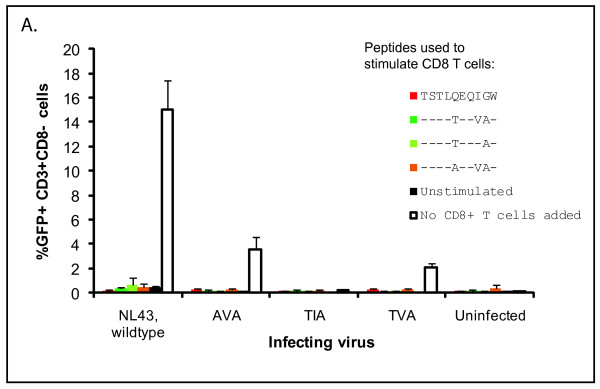
**Unstimulated CD8^+ ^T cells from ES8 block infection by autologous and wild type virus (described in Figure 2a) as effectively as cells stimulated with autologous or wild type TW10 peptides**. CD4^+ ^T cells from the patient were infected via spinoculation with wild type NL43 and NL43-based viruses expressing autologous TW10 epitopes, as described in Figure 2a. Cells were then co-cultured with CD8^+ ^T cells from ES8 which were either unstimulated or had been stimulated with the TVA, TIA, or AVA peptides for four days. Co-culture with infected cells was for seven days. Results shown are averages of triplicate experiments with error bars indicating standard deviation.

To explore the strength of the patient's CTL response to autologous and wild type TW10, we measured the ability of CD8^+ ^T cells to suppress the replication competent pseudo-type viruses described in Figure 2a. We stimulated PBMC from ES8 with three autologous TW10 variants (TSTL**A**EQ**VA**W, TSTL**T**EQI**A**W, TSTL**T**EQ**VA**W) as well as wild type TW10 for 6 days, and then isolated CD8^+^T cells from these PBMCs. We then infected PHA activated CD4^+^T cells from ES8 with the replication competent viruses constructed to express these TW10 variants, and co-cultured the infected cells with each CD8^+^T cell population that had been stimulated with the four peptides, or with unstimulated CD8^+^T cells. As shown in Figure 4a, CD8^+^T cells that were not pre-incubated with HIV peptides were as efficient as CD8+ T cells that had been stimulated with peptides in inhibiting replication of both wild type virus and escape mutants. This highlights once again the remarkable ability of CTL from this ES to suppress viral replication of both autologous and non-autologous virus without exogenous stimulation.

While cross-reactivity between wild type and mutant epitopes has been documented, *de novo *responses to mutated HIV-1 epitopes are rare. With regard to HLA-B*57-restricted epitopes, one study suggested that de novo CTL responses to autologous epitope variants emerged more frequently in children than in adults [25]. However, the emergence of de novo responses to mutant TW10 variants has been observed by one group in both CP and LTNPs[12]. We [4] and others [13] have reported *de novo *responses to escape mutants in ES, but these studies have looked only at IFN-γ secretion. IFN-γ is not the ideal measure of an effective CTL response and does not correlate with immune protection [20-22]. In contrast, HIV-specific CD8^+ ^T cells that proliferate in response to antigen have been associated with perforin secretion and effective immune control of viral replication [24]. Furthermore the ability of primary CD8^+ ^T cells to inhibit HIV replication has been shown to be a correlate of immunity [26,27]. We show here that CD8^+ ^T cells from this patient proliferate effectively and inhibit replication of multiple autologous escape variants. This may explain his continued elite control.

In this study we describe an ES whose immune system has selected for seven rare TW10 variants in Gag. The development of these variants was apparently due to the selective pressure placed on the virus by the CTL response to the wild type TW10 epitope, as shown in Figure 3c. These mutants have dramatically reduced viral fitness and are continuing to evolve over time. It is not clear why the T242N mutation never developed in this patient, analysis of TW10-specific T cell receptors in patients who develop rare alternate mutations in this epitope may be revealing. The continued evolution in this patient is striking as the typical ES has one epitope variant present in the plasma which remains constant [6], but the presence of multiple variants may be partially due to the fact that this patient has maintained higher viral loads (mean of 26 copies/ml) than the other ES in our cohort [28]. ES8 has developed de novo CTL responses to the variants which proliferate in response to antigen and suppress viral replication. Further studies will be needed to determine whether the induction of de novo proliferative suppressive CTL responses is feasible and protective.

## Competing interests

The authors declare that they have no competing interests.

## Authors' contributions

KAO carried out the molecular genetic studies, sequence analysis, and drafted the manuscript. RH provided patient samples. RFS participated in study design and helped to draft the manuscript. JNB conceived of the study, participated in its design and coordination, and helped to draft the manuscript. All authors read and approved the final manuscript.
